# Income-related equity in inpatient care utilization and unmet needs between 2013 and 2018 in Tibet, China

**DOI:** 10.1186/s12939-023-01889-4

**Published:** 2023-05-10

**Authors:** Cidan Zhuoga, Zhaxi Cuomu, Shunping Li, Lei Dou, Chaofan Li, Zhaxi Dawa

**Affiliations:** 1 Medical College of Tibet University, Lhasa, 850000 China; 2 Center of Tibetan Studies (Everest Research Institute), Tibet University, Lhasa, 850000 China; 3 Centre for Health Management and Policy Research, School of Public Health, Cheeloo College of Medicine, Shandong University, Jinan, 250012 China; 4 NHC Key Lab of Health Economics and Policy Research, Shandong University, Jinan, 250012 China; 5 Center for Health Preference Research, Shandong University, Jinan, 250012 China; 6 High Altitude Health Science Research Center, Tibet University, Lhasa, 850000 China

**Keywords:** Equity, Inpatient care utilization, Unmet needs, Tibet, Concentration index

## Abstract

**Background:**

Providing equitable access to health care for all populations is an important sustainable development goal. China has made significant progress in achieving equity in healthcare utilization. However, research on equity in healthcare utilization in Tibet is sparse. This study aims to evaluate changes in income-related inequity in inpatient care utilization and unmet needs between 2013 and 2018 among the Tibetan population and identify the inequity source.

**Methods:**

Data for this cross-sectional study were obtained from the fifth and sixth waves of the National Health Services Survey in 2013 and 2018. After excluding observations with missing values for key variables, 11,092 and 10,397 respondents were included in this study, respectively. The outcome variables of interest were inpatient service utilization and unmet hospitalization needs. The concentration index and horizontal inequity index (HI) were used to assess income-related inequity. Non-linear decompositions were performed to identify the main contributors to inequity. In the decomposition method, need variables included sex, age, chronic diseases, and the EuroQol-Visual Analog Scale; non-need variables consisted of income, education, employment status, marital status, and health insurance schemes.

**Results:**

The probability of inpatient care utilization increased from 6.40% in 2013 to 8.50% in 2018. The HI for inpatient care utilization was 0.19 (*P* < 0.001) in 2013, whereas it decreased to 0.07 (*P* < 0.001) in 2018. The contribution of income to inequity in inpatient care utilization decreased from 87.09% in 2013 to 59.79% in 2018. As for unmet inpatient care needs, although its probability increased from 0.76 to 1.48%, the percentage of reasons for financial hardship decreased from 47.62 to 28.57%. The HI for unmet hospitalization need was − 0.07 in 2013 and − 0.05 in 2018, and neither was statistically significant. The New Rural Cooperative Medical Scheme made majority contributions to promote equity in unmet hospitalization need. Moreover, the female respondents reporting low EuroQol-Visual Analog Scale scores and patients with chronic disease were not only more likely to seek for inpatient care, but also have more unmet need than the reference groups.

**Conclusions:**

The inequity in inpatient care utilization in Tibet narrowed from 2013 to 2018, and there was no inequity in unmet hospitalization needs in 2013 and 2018. Income and the New Rural Cooperative Medical Scheme are the main drivers of equity promotion. To promote access to inpatient care utilization and decrease the probability of unmet hospitalization need in future, policymakers should target high-need residents in Tibet to improve accessibility, availability, and acceptability.

**Supplementary Information:**

The online version contains supplementary material available at 10.1186/s12939-023-01889-4.

## Background

Achieving universal health coverage—which includes providing equitable access to high-quality healthcare for all populations—is an important target of the Sustainable Development Goals proposed by the United Nations in 2015 [1]. During the past years, equity in healthcare utilization has drawn much attention from both developed and developing countries [2, 3]. In the literature, equality in healthcare utilization means there is no difference in utilization by socioeconomic status [4], and the principles of healthcare equity imply that it should be allocated based on medical needs, irrespective of income, race, education, or region of residence [5]. Empirical research usually focuses on horizontal equity in healthcare utilization, which states that individuals with equal needs should be treated equally irrespective of socioeconomic factors [5]. In practice, horizontal equity in healthcare utilization can be reflected by examining differences in healthcare across socioeconomic factors after adjusting for differences in needs [6].

China, the largest developing country in the world, has implemented a new round of health system reforms since 2009 to provide equal access to primary healthcare for the whole population [7]. In the first reform phase (2009–2011), the Chinese government reconstructed the primary healthcare delivery system, expanded health insurance coverage, and quadrupled financial investments in health to improve availability and affordability [8, 9]. China achieved universal health insurance coverage for 1.3 billion people in 2011 by expanding the Urban Employee Basic Medical Insurance (UEBMI) for the employed, the New Rural Cooperative Medical Scheme (NRCMS) for rural residents, and the Urban Residents Basic Medical Insurance (URBMI) for the unemployed, children, and students in urban areas [10]. More than 95% of the Chinese population became insured, which was much higher than the 50% in 2005 [10]. In the second phase (2012 onwards), the Chinese government prioritized public hospital reform and provider payments to control health expenditure growth [9]. Until 2019, China has made substantial achievements in improving equal access to healthcare [9].

The Tibet Autonomous Region, located in southwestern China, is a mountainous region with high altitudes of over 4,000 m, covering more than 1.2 million square kilometers, which accounts for approximately one-eighth of China’s total territorial area [11]. Its population was approximately 3.65 million in 2020 [3]. Due to the adverse natural environment, living styles, low socioeconomic development, and poor healthcare resources, the health status of the residents in Tibet was worse than those in other regions [12]. The Chinese have focused on improving health status and access to healthcare for the Tibetan. During the past several decades, the life expectancy in Tibet has increased from 35.5 years in 1951 to 72.2 years in 2021 [13]. However, the residents still face many barriers to healthcare, including low economic development, dispersed living, poor transportation, low density of health resources, inadequate communication skills, and health literacy [14, 15]. Promoting equitable access to healthcare in Tibet remains a great challenge.

The health system in Tibet is a primary care-based system, which consists of township health centers, county hospitals, and prefecture and above hospitals [16]. Township health centers mainly provide outpatient care and hospitals provide the majority of inpatient care. In 2018, 59% of inpatient care was concentrated in county hospitals, 28% was provided by prefecture and above hospitals, and only 5.5% was provided by township health centers [17]. Each hospital covers a wide range of geographical areas and patients usually face accessibility barriers to access inpatient care [18]. Regarding availability, the density of health professionals and ward beds was 5.5 and 4.9, respectively, per 1,000 persons in 2018 in Tibet, which is lower than the national level (6.8 and 6.0, respectively) [19]. In contrast, the affordability of healthcare in Tibet was much better than that in other regions. The NRCMS covered 100% of the rural and nomadic population in Tibet, and reimbursed approximately 85% of inpatient expenditures [20]. The share of out-of-pocket (OOP) expenses in total health expenditure (THE) in Tibet was 5.62% in 2018, which is much lower than the national average of 28.61% [21]. The details of healthcare availability and affordability in Tibet are reported in Supplementary Table 1.

Tibet has low availability and accessibility and high affordability of healthcare. Previous studies had found that the probability of inpatient care utilization among the Tibetan was lower than that among other regions [17, 22]. In addition, Mu and Gao observed slight inequality in health-related quality of life and health resource allocation among Tibetans [15, 18]. However, studies on the equity in inpatient care utilization in Tibet are scarce. In order to provide sufficient evidences for health policy making, we have to empirically examine the magnitude of inequity in healthcare utilization and identify the source of inequity. Moreover, little research has been conducted on unmet healthcare needs among Tibetans. Unmet healthcare needs are defined as differences between the actual health services received by individuals and those deemed necessary to treat a particular disease by a physician [23]. It has received increasing attention from policymakers, and has been recognized as an important indicator for monitoring equity in healthcare utilization [24].

To address this research gap, our study aimed to examine the equity of inpatient care utilization and unmet needs among Tibetans. Furthermore, we analyze the change in equity between 2013 and 2018 and identify the source of inequity to provide empirical evidence for equity-oriented health policy making.

## Methods

### Data sources

Data for this cross-sectional study were obtained from the fifth and sixth waves of the National Health Services Survey (NHSS) in Tibet in 2013 and 2018. The NHSS is conducted every five years, and the fifth and sixth surveys were conducted in 2013 and 2018. The questionnaire covered a wide range of themes such as household characteristics, demographics, socioeconomic status, health status, health care utilization, health expenditure, and health insurance schemes. Residents were selected using multiple-stage stratified cluster random sampling methods. In the first stage, 24 counties were selected in 2013 and 25 counties were selected in 2018 using a stratified sampling method according to 10 socioeconomic and demographic variables, including the percentage of primary industry in the economy, crude mortality, infant mortality, and the percentage of adults aged 65 years and above. In the second stage, 60 towns were sampled in 2013 and 59 towns were sampled in 2018 using the random cluster method according to population size. In the third stage, 155 villages or communities were selected in 2013 and 159 villages or communities were selected in 2018 using the random cluster method. In the previous three stages, the majority of the counties, towns, and villages sampled in 2018 were the same as those sampled in 2013. In the fourth stage, 4,140 households were randomly sampled in 2013 and 4,232 households were randomly sampled in 2018 from villages or communities, and residents aged 16 years were interviewed individually. Household members younger than 16 years were excluded in this study because the NHSS questionnaire did not collect information regarding their self-rated health status and chronic disease. Finally, 11,336 respondents in 2013 and 10,431 respondents in 2018 were surveyed by trained interviewers using face-to-face computer-assisted personal interviewing techniques. After excluding observations with missing values for key variables, 11,092 and 10,397 respondents were included in the analysis. The response rates in 2013 and 2018 were 97.85% and 99.67%, respectively. As previous studies have pointed out, the sampled respondents of the NHSS were representative of the whole population [12, 25]. The respondents sampled in 2013 were not followed up on in 2018; thus, the respondents were not the same for both years.

### Outcome variables

The outcome variables of interest were inpatient service utilization and unmet hospitalization needs. The measurement of inpatient service utilization was based on the following question: “During the past year, did you have inpatient visits because of disease, injury, or physical examination?” The respondents’ answers were dichotomous variables: 0 for “No” and 1 for “Yes.” Unmet hospitalization need was measured based on the following question: “During the past year, did the doctor refer you to hospitalization services but you did not seek them?” The answer was coded as a dummy variable (0 = no; 1 = yes). If the respondents answered “Yes,” they were asked to explain why they did not seek hospitalization services as per the doctor’s suggestion. The response options were as follows: (1) I thought it was not necessary; (2) I thought that there was no effective treatment care; (3) Financial hardship; (4) I thought the hospitalization services were bad; (5) I had no time; (6) There was no spare bed in the hospital; (7) The restriction of health insurance reimbursement policies; and (8) Other reasons. As the frequency of some reasons was very low, eight reasons were coded into four groups: unnecessary, financial hardship, no time, and other reasons.

### Independent variables

Following the guidelines for analyzing income-related health equity, the independent variables were selected according to previous literature on factors associated with inpatient utilization and unmet hospitalization need and classified into two groups: need and non-need variables [6, 24, 26]. Need variables included sex, age, the EuroQol-Visual Analog Scale (EQ-VAS), and the number of chronic diseases. Sex is a dummy variable, and age was categorized in to four groups: 16–29, 30–44, 45–59, and 60+. The EQ-VAS was measured by the following question: “What score represents your health today? Please rate your health status, with 0 representing worst and 100 representing best.” The scores were displayed using a horizontal 11 cm line labeled 0, 10, …, 100. The EQ-VAS is a reliable and valid tool to assess an individual’s functioning and well-being, and is feasible for measuring health status among Chinese individuals [25, 27]. The number of self-reported chronic diseases was measured by the following question: “How many chronic diseases, such as hypertension, diabetes, or other physician-diagnosed diseases, did you experience during the past six months?” Respondents’ answers were categorized into four groups: no chronic disease, one, two, and three and above.

The non-need variables included income, marital status, employment status, education, and health insurance schemes. The income was measured by per capita household income, which was calculated by dividing self-reported household income by the number of household members. It was transported to the value of 2018 by multiplying consumer price index. Marital status was grouped into three types: unmarried, married, and divorced. Employment status was also coded into three groups: employed, retired, and unemployed. Education was divided into four levels: below primary school, primary school, junior middle school, and senior middle school. Health insurance schemes included the NRCMS, URBMI, UEBMI, and other medical insurance schemes. Respondents were asked whether they enrolled in these health insurance schemes individually. The answers were coded as dummy variables: 0 for “No” and 1 for “Yes.”

### Statistical analysis

First, we conducted descriptive statistics to summarize the respondents’ basic characteristics. The frequency and percentage were used to describe the characteristics of the categorical variables, and the chi-square test was used to examine the differences between 2013 and 2018. Mean and standard deviation were used to describe the characteristics of continuous variables, and the Kruskal–Wallis test was performed to analyze differences between 2013 and 2018 because of their non-normal distribution.

Second, the concentration index (CI) was used to measure inequality in healthcare utilization, and the horizontal inequity index (HI) was used to measure the income-related inequity. The CI is a specific approach to measure and compare income-related inequality in healthcare utilization [28]. In this study, CI was calculated using the “convenient regression” approach and obtained from the “conindex” Stata command [6, 28]:

1 $$2{\sigma }_{r}^{2}\left(\frac{{h}_{i}}{\mu }\right)=\alpha +\beta {r}_{i}+{\epsilon }_{i}$$

where ${\sigma }_{r}^{2}$ is the variance of the rank of per capita household income, ${h}_{i}$ is healthcare utilization for individual *i*, $\mu$ is the mean, ${r}_{i}$ is the fractional rank of per capita household income for individual *i*, coefficient $\beta$ is equivalent to CI. CI ranges from µ-1 to 1-µ, with positive (negative) value indicating pro-rich (poor) inequality in healthcare utilization and zero meaning equality [6].

Horizontal inequity index was captured using two steps. In the first step, need-standardized healthcare utilization was calculated based on the logistic regression model following the procedure proposed by Wagstaff [6], in which the need variables included sex, age, EQ-VAS, and chronic disease. In the second step, CI for need-standardized healthcare utilization, namely HI, was calculated using the “conindex” command. Positive (negative) value of HI indicated pro-rich (poor) inequity in healthcare utilization and zero means horizontal equity.

Third, to identify the source of income-related inequality in healthcare utilization, we decomposed CI using logit model. The reason for using logit model was that the healthcare utilization variable was binary and had leptokurtic distribution [29]. In this model,

2 $${y}_{i}=G\left(\alpha +{\sum }_{j}{\beta }_{i}{x}_{ji}+{\sum }_{k}{\gamma }_{j}{z}_{ki}\right)+{\epsilon }_{i}$$

where *y* is healthcare utilization, G takes a particular form for the logit, ${x}_{j}$ is a need variable, ${z}_{k}$ is a non-need variable, *i* represents an individual, and $\epsilon$ denotes residual terms. Robust standard errors clustering on the household level were used to correct for heteroscedasticity and potential cluster sampling. The decomposition results of the CI for healthcare utilization can be written as:

3 $$C={\sum }_{j}({\beta }_{j}^{m}{\stackrel{-}{x}}_{j}/\mu ){C}_{j}+{\sum }_{k}({\gamma }_{k}^{m}{\stackrel{-}{z}}_{k}/\mu ){C}_{k}+G{C}_{u }/u$$

Where C is the CI for healthcare utilization; $\mu$ is the mean of healthcare utilization; ${\beta }^{m}$ and ${\gamma }^{m}$ are the marginal effects of need and non-need variables that could be captured from the logistic regression model; $\stackrel{-}{x}$ and $\stackrel{-}{z}$ are means of need and non-need variables, respectively; ${C}_{j}$ and ${C}_{k}$ are CI for need and non-need variables, respectively; $\left({\beta }^{m}\stackrel{-}{x}/\mu \right)C$ is the contribution of the need or non-need variable to income-related inequality in healthcare utilization; and $G{C}_{u }/u$ is the contribution of residual terms. In the logistic regression model, per capita household income was transformed into a logarithmic form, consistent with the previous literature [30]. All statistical analyses were conducted using STATA 15.0 [31].

## Results

Table 1 displays the basic characteristics of the sampled residents. Approximately 47% of respondents were men, and about 60% of the respondents were middle-aged between 30 and 59. The mean EQ-VAS score decreased significantly from 72 to 69 between 2013 and 2018 (*P* < 0.001), whereas the probability of chronic disease increased from 33.5 to 41% (*P* < 0.001). As for the non-need variables, per capita household income significantly increased by approximately 1,817 Yuan RMB (*P* < 0.001). The majority of respondents (approximately 75%) were married, and more than 80% were employed. The percentage of respondents with education lower than the primary school level decreased by 7.2%, and the percentage of respondents with a primary school, junior middle school, and senior middle school education increased by approximately 1.5 − 2%. More than 80% of the respondents were enrolled in the NRCMS, and approximately 15% of them were enrolled in the URBMI. The proportion of respondents enrolled in the UEBMI and other medical insurance was about 5%. In total, approximately 99% of respondents were covered by some type of health insurance scheme, and some were enrolled in two or more health insurance types.

**Table 1:** Descriptive statistics

Variables	Category	2013, *N* (%)	2018, *N* (%)	Chi square	*P*
Total		11,092	10,397	-	-
Outcome variables					
Inpatient care utilization		710 (6.40)	884 (8.50)	34.51	< 0.001
Unmet inpatient care need		84 (0.76)	154 (1.48)	25.68	< 0.001
Reason for unmet inpatient care need	Unnecessary	5 (5.95)	23 (14.94)	13.68	0.003
	Financial hardship	40 (47.62)	44 (28.57)		
	No time	15 (17.86)	21 (13.64)		
	Others	24 (28.57)	66 (42.86)		
Need variables					
Sex	Male	5223 (47.09)	4878 (46.92)	0.06	0.802
	Female	5869 (52.91)	5519 (53.08)		
Age, years	16–29	2932 (26.43)	1983 (19.07)	176.11	< 0.001
	30–44	3443 (31.04)	3406 (32.76)		
	45–59	2944 (26.54)	3248 (31.24)		
	60+	1773 (15.98)	1760 (16.93)		
EQ-VAS, mean (SD)		71.94 (16.21)	69.14 (19.44)	62.03	< 0.001
Chronic disease	No	7380 (66.53)	6136 (59.02)	433.20	< 0.001
	1 kind	1657 (14.94)	2719 (26.10)		
	2 kinds	1328 (11.97)	1074 (10.33)		
	3 kinds and above	727 (6.55)	468 (4.50)		
Non-need variables					
Per capita household income (RMB yuan), mean (SD)		9319.70 (14286.14)	11136.81 (19475.73)	267.85	< 0.001
Marital status	Unmarried	1891 (17.05)	1491 (14.34)	29.78	< 0.001
	Married	8209 (74.01)	7932 (76.29)		
	Divorced	992 (8.94)	974 (9.37)		
Employment status	Employed	9130 (82.31)	8427 (81.05)	6.35	0.042
	Retired	222 (2.00)	207 (1.99)		
	Unemployed	1740 (15.69)	1763 (16.96)		
Education level	Lower than primary school	6151 (55.45)	5018 (48.26)	116.59	< 0.001
	Primary school	3299 (29.74)	3480 (33.47)		
	Junior middle school	1033 (9.31)	1172 (11.27)		
	Senior middle school	609 (5.49)	727 (6.99)		
NRCMS	No	1871 (16.87)	1612 (15.50)	7.35	0.007
	Yes	9221 (83.13)	8785 (84.50)		
URBMI	No	9482 (85.49)	8759 (84.25)	6.43	0.011
	Yes	1610 (14.51)	1638 (15.75)		
UEBMI	No	10,852 (97.84)	10,045 (96.61)	29.91	< 0.001
	Yes	240 (2.16)	352 (3.39)		
Other medical insurance	No	11,064 (99.75)	10,158 (97.70)	183.14	< 0.001
	Yes	28 (0.25)	239 (2.30)		

SD, standard deviation; EQ-VAS, EuroQol Visual Analogue Scale; NRCMS, New Rural Cooperative Medical Schemes; URBMI, Urban Residents Basic Medical Insurance; UEBMI, Urban Employees Basic Medical Insurance

Among the respondents, the probability of inpatient care utilization increased from 6.40% to 2013 to 8.50% in 2018. Although the probability of unmet inpatient care needs increased from 0.76 to 1.48%, the percentage of reasons for financial hardship decreased from 47.62 to 28.57%. There were significant differences in the probability of inpatient care utilization (*P* < 0.001), unmet needs (*P* < 0.001), and the distribution of reasons for unmet needs (*P* = 0.003).

Table 2 shows the CI and HI for inpatient care utilization and unmet needs. The CI for inpatient care utilization in 2013 and 2018 was 0.18 (*P* < 0.001) and 0.04 (*P* = 0.053), respectively. After adjusting for need variables, the HI for inpatient care utilization was significant at 0.19 (*P* < 0.001) in 2013, whereas it decreased to 0.07 (*P* < 0.001). The differences in HI between the two years were significant at the 0.001 level. For unmet inpatient care needs, the CI increased from − 0.09 to -0.07, and the HI increased from − 0.07 to -0.05. However, none of the CI and HI scores for unmet needs and their differences between the two years were significant.

**Table 2:** Concentration index and horizontal inequity in inpatient care utilization and unmet inpatient care need

Outcome variables	Concentration index		Horizontal inequity	
	Index value (Robust SE)	*P*	Index value (Robust SE)	*P*
Inpatient care utilization				
2013	0.18 (0.02)	0.001	0.19 (0.02)	< 0.001
2018	0.04 (0.02)	0.053	0.07 (0.02)	< 0.001
Differences between 2013 and 2018	-0.14 (0.03)	< 0.001	-0.12 (0.03)	< 0.001
Unmet inpatient care need				
2013	-0.09 (0.07)	0.164	-0.07 (0.07)	0.282
2018	-0.07 (0.05)	0.188	-0.05 (0.05)	0.309
Differences between 2013 and 2018	0.02 (0.08)	0.764	0.02 (0.08)	0.815

SE, standard error

Table 3 displays the decomposition of CI for inpatient care utilization in 2013 and 2018. The need variables were all significantly correlated with inpatient care utilization over the two years. Women and respondents with chronic diseases were more likely to use inpatient care, whereas older respondents and respondents reporting higher EQ-VAS scores were less likely to seek inpatient care. Among the non-need variables, the log of income was significantly positively associated with inpatient care utilization in 2013 (β = 0.831, *P* < 0.001), but not significant in 2018 (β = 0.123, *P* > 0.05). Income was the major source of inequality in inpatient care utilization in 2013, which contributed to approximately 87% of the inequity. Moreover, referring to respondents without health insurance coverage, those enrolled in URBMI and UEBMI were significantly less likely to have inpatient care in 2013, but they were not significantly less likely to have inpatient care in 2018. The URBMI and UEBMI made negative contributions to pro-rich inequality in inpatient care utilization in 2013, which were as high as -19.82% and − 7.33%, respectively. The retired were more likely to use inpatient care than the employed, and respondents with a primary school education had a higher likelihood of seeking inpatient care than those with an education lower than primary school.

**Table 3:** Decomposition of concentration index for inpatient care utilization among Tibetan in 2013 and 2018

Inpatient care utilization	2013					2018				
	CI	β	Elasticity	Contribution	Contribution (%)	CI	β	Elasticity	Contribution	Contribution (%)
Need variables				-0.010	-5.75				-0.023	-59.37
Female (ref. Male)	-0.008	0.522^***^	0.198	-0.002	-0.88	-0.004	0.590^**^	0.236	-0.001	-2.70
Age 30–44 (ref. 16–29)	0.007	-0.464^***^	-0.103	-0.001	-0.43	0.003	-0.813^***^	-0.201	-0.001	-1.52
Age 45–59	0.005	-0.617^***^	-0.117	-0.001	-0.31	0.042	-1.174^***^	-0.276	-0.012	-30.26
Age 60+	-0.027	-0.686^***^	-0.079	0.002	1.21	-0.041	-0.885^***^	-0.113	0.005	11.94
EQ-VAS	0.011	-0.022^***^	-1.157	-0.013	-7.20	0.022	-0.015^***^	-0.785	-0.017	-44.14
Chronic disease: 1 kind (ref. No)	-0.015	0.680^***^	0.073	-0.001	-0.62	0.017	0.568^***^	0.112	0.002	4.97
2 kinds	0.005	0.701^***^	0.060	0.000	0.17	0.009	1.046^***^	0.081	0.001	1.96
3 kinds and above	0.069	1.258^***^	0.059	0.004	2.32	0.004	1.200^***^	0.041	0.000	0.38
Non-need variables				0.142	79.92				0.045	116.35
Log of income	0.070	0.831^**^	2.218	0.154	87.09	0.066	0.123	0.350	0.023	59.79
Married (ref. Unmarried)	0.016	0.545^***^	0.289	0.005	2.62	0.013	0.822^***^	0.473	0.006	15.70
Divorced	-0.097	-0.150	-0.010	0.001	0.53	0.000	0.675^***^	0.048	0.000	-0.05
Retired (ref. Employed)	0.620	0.933^***^	0.013	0.008	4.68	0.616	0.616^**^	0.009	0.006	14.71
Unemployed	0.035	0.241^*^	0.027	0.001	0.54	-0.083	0.017	0.002	0.000	-0.47
Primary school (ref. Lower than primary school)	0.013	-0.029	-0.006	0.000	-0.05	0.006	0.250^**^	0.063	0.000	1.03
Junior middle school	0.089	0.116	0.008	0.001	0.39	0.108	0.032	0.003	0.000	0.76
Senior middle school	0.303	-0.170	-0.007	-0.002	-1.14	0.295	0.190	0.010	0.003	7.65
NRCMS (ref. No)	-0.064	-0.578	-0.344	0.022	12.41	-0.053	-0.044	-0.028	0.002	3.89
URBMI (ref. No)	0.260	-1.300^***^	-0.135	-0.035	-19.82	0.109	-0.133	-0.016	-0.002	-4.45
UEBMI (ref. No)	0.698	-1.200^**^	-0.019	-0.013	-7.33	0.621	0.361	0.009	0.006	14.76
Other health insurance (ref. No)	0.000	0.001	0.507	0.000	0.00	0.216	0.314	0.005	0.001	3.04
Residual				0.046	25.83				0.017	43.01

^*^, *P* < 0.05; ^**^, *P* < 0.01; ^***^, *P* < 0.001.CI, concentration index; EQ-VAS, EuroQol Visual Analogue Scale; NRCMS, New Rural Cooperative Medical Schemes; URBMI, Urban Residents Basic Medical Insurance; UEBMI, Urban Employees Basic Medical Insurance

Table 4 shows the decomposition of the CI for unmet inpatient care needs. Among the need variables, sex, EQ-VAS score, and the number of chronic diseases were significantly related to unmet needs. Female respondents and patients with one or more kinds of chronic diseases were more likely to have unmet needs than the reference group, whereas respondents with high EQ-VAS scores were less likely to report unmet need. Notably, income was not significantly associated with unmet inpatient care needs in either 2013 or 2018 (*P* > 0.05). Respondents with junior middle school (β=-1.030, *P* < 0.05) education were less likely to have unmet needs than the reference group in 2018. Furthermore, referring to the respondents without health insurance coverage, the adults enrolled in the NRCMS were less likely to have unmet needs (β=-2.104, *P* < 0.01) in 2013. The NRCMS made positive contributions to the CI for unmet needs which accounted for 73.37% in 2013 and 35.97% in 2018.

**Table 4:** Decomposition of concentration index for unmet inpatient care need among Tibetan in 2013 and 2018

Inpatient care utilization	2013					2018				
	CI	β	Elasticity	Contribution	Contribution (%)	CI	β	Elasticity	Contribution	Contribution (%)
Need variables				-0.012	12.72				-0.010	14.79
Female (ref. Male)	-0.008	0.826^***^	0.261	-0.002	2.27	-0.004	0.508^**^	0.193	-0.001	1.29
Age 30–44 (ref. 16–29)	0.007	-0.367	-0.068	0.000	0.55	0.003	-0.566	-0.132	0.000	0.59
Age 45–59	0.005	-0.596	-0.094	0.000	0.49	0.042	-0.693^*^	-0.155	-0.007	9.90
Age 60+	-0.027	-0.825	-0.079	0.002	-2.35	-0.041	-0.446	-0.054	0.002	-3.33
EQ-VAS	0.011	-0.028^***^	-1.204	-0.013	14.60	0.022	-0.007	-0.368	-0.008	12.11
Chronic disease: 1 kind (ref. No)	-0.015	1.411^***^	0.126	-0.002	2.08	0.017	0.870^***^	0.162	0.003	-4.22
2 kinds	0.005	1.070^**^	0.076	0.000	-0.43	0.01	1.243^***^	0.092	0.001	-1.29
3 kinds and above	0.069	1.500^***^	0.059	0.004	-4.49	0.004	1.430^***^	0.046	0.000	-0.25
Non-need variables				-0.017	18.91				-0.031	46.74
Log of income	0.070	-0.291	-0.647	-0.045	49.49	0.066	-0.228	-0.619	-0.041	61.72
Married (ref. Unmarried)	0.016	0.454	0.201	0.003	-3.55	0.013	0.291	0.159	0.002	-3.08
Divorced	-0.097	0.769	0.041	-0.004	4.37	0.000	0.149	0.010	0.000	0.01
Retired (ref. Employed)	0.620	0.316	0.004	0.002	-2.58	0.616	-0.436	-0.006	-0.004	5.77
Unemployed	0.035	-0.153	-0.014	-0.001	0.55	-0.083	-0.258	-0.031	0.003	-3.90
Primary school (ref. Lower than primary school)	0.013	0.413	0.073	0.001	-1.09	0.006	-0.053	-0.013	0.000	0.12
Junior middle school	0.089	0.773	0.043	0.004	-4.22	0.108	-1.030^*^	-0.083	-0.009	13.55
Senior middle school	0.303	0.400	0.013	0.004	-4.37	0.295	-0.373	-0.019	-0.005	8.31
NRCMS (ref. No)	-0.064	-2.104^**^	-1.044	0.067	-73.37	-0.053	-0.739	-0.446	0.024	-35.97
URBMI (ref. No)	0.260	-1.383	-0.120	-0.031	34.22	0.109	0.358	0.040	0.004	-6.62
UEBMI (ref. No)	0.698	-2.110	-0.027	-0.019	20.93	0.621	-0.051	-0.001	-0.001	1.15
Other health insurance (ref. No)	0.507	1.769	0.003	0.001	-1.49	0.216	-1.061	-0.017	-0.004	5.69
Residual				-0.062	68.37				-0.025	38.47

^*^, *P* < 0.05; ^**^, *P* < 0.01; ^***^, *P* < 0.001

CI, concentration index; con, contribution; EQ-VAS, EuroQol Visual Analogue Scale; NRCMS, New Rural Cooperative Medical Schemes; URBMI, Urban Residents Basic Medical Insurance; UEBMI, Urban Employees Basic Medical Insurance

## Discussion

This cross-sectional study examined the change of equity of inpatient care utilization and unmet needs in Tibet in 2013 and 2018 and identified the inequity source using two waves of the NHSS. Our findings indicate that pro-rich inequity in inpatient care utilization attenuated from 2013 to 2018, and there was no inequity in unmet needs. Moreover, the main reason for unmet needs switched from financial hardships to others. The income and health insurance schemes are the main drivers of equity promotion in Tibet.

First, the probability of inpatient care utilization among Tibetans was lower than that among the national population (9.0% in 2013 and 13.7% in 2018, respectively) [32, 33]. This may be due to the poor availability and accessibility of hospital services in Tibet compared to other regions; the population density, density of physicians, and ward beds in Tibet were the lowest [21]. Moreover, predisposing factors such as low health literacy, health risk perception, and religious belief may also decrease the likelihood of seeking inpatient care among the Tibetan [17, 22]. Specially, some old residents in Tibet preferred praying to Buddha rather than going to the hospital when they felt ill [34].

Regarding equity, we found that inpatient care utilization was unequally concentrated among the rich. Even if the need factors were adjusted for, an uneven distribution was retained. This result illustrates that the poor in Tibet did not receive equal treatment for equal needs. In other words, there was inequitable hospital visit utilization in Tibet. Li also found pro-rich inequity in inpatient care utilization among the entire population of China in 2013 [35]. The HI of inpatient care utilization for the national population was 0.082 in 2013, which was lower than that for Tibet [35]. This illustrates that inequity in Tibet was more serious than that in the entire population. According to the regression model and decomposition results, income itself was the main driver of pro-rich inequity, making approximately 87% of the contribution to inequity. In line with our findings, Li also reported that economic status contributed 93% to inequity in the probability of inpatient service utilization among middle-aged and older adult individuals in 2013 [36].

Moreover, the CI for inpatient care utilization were not significant in 2018, and HI decreased to 0.07 in 2018. This demonstrates that inequity in inpatient care utilization narrowed drastically from 2013 to 2018. A similar trend has also been observed, in that, pro-rich inequity in inpatient care utilization in China has narrowed substantially or disappeared since 2010 [9, 37]. Income itself is the main promoter of equity. First, the relationship between income and inpatient care utilization was significant in 2013, but non-significant in 2018. Second, the CI for income decreased from 0.070 to 2013 to 0.066 in 2018, indicating that income inequality had decreased. Zhou found that narrowing the income gap promotes equity in healthcare [26]. Therefore, the contribution of income to the CI decreased from 87% in 2013 to 60% in 2018. The increase in income level and narrowing of income disparity were the main drivers promoting equity in inpatient care utilization.

Second, the probability of unmet inpatient care needs in Tibet was lower than that in other regions, but increased from 2013 to 2018. As Zhou reported, the probability of unmet hospitalization needs among the population in the Jiangsu province was 1.05% in 2013 and 1.77% in 2018, respectively [38]. A possible explanation for the low probability of unmet inpatient care needs in Tibet is that the affordability of hospital services was very high. The proportion of OOP in THE was the lowest among all the provinces in China, and NRCMS reimbursed the majority of inpatient expenditure. The share of OOP in THE was positively associated with the probability of unmet needs [39]. Thus, financial hardship was not the main reason for unmet needs in Tibet, and residents had high affordability of hospital services, especially in 2018. In contrast, financial hardship is still the most common reason for unmet inpatient care needs among populations in other regions [24, 32, 33, 38, 40].

The explanation of increment of probability of unmet hospitalization needs may be lack of health literacy. As Guosheng reported, the health literacy and education level among Tibetan residents was lower than that among populations living in other provinces [41]. Previous studies had found that residents with a lack of health literacy were more likely to have unmet healthcare needs [42]. When physicians recommended patients with inadequate health literacy to have hospitalization services, they were more likely to forgo healthcare utilization. As the results displayed, the frequency and proportion of residents in Tibet choosing “unnecessary” and “no time” as main reason for unmet need had increased from 2013 to 2018. This phenomenon may illustrate that inadequate health literacy is becoming a prominent barrier to equitable inpatient care utilization among the Tibetan.

Concerning equity in unmet needs, although the CI was negative in 2013 and 2018, neither was significant. Even after adjusting for the need factors, the HI was still non-significant. The decomposition results and regression models also showed that income was not correlated with unmet needs adjustment for need or other non-need variables. Thus, we found no inequity in unmet needs among Tibetans. This finding was in contrast with a previous study, which found that the poor were more likely to have unmet needs than the rich after adjusting for other demographic and need variables [38, 40]. According to the decomposition results, the NRCMS decreased the likelihood of unmet needs and contributed to equity. As Zhou suggested, health insurance coverage can reduce the prevalence of unmet needs [38]. Therefore, the NRCMS should be the main factor in promoting equity in unmet needs.

Third, we found that the number of diseases and EQ-VAS scores were closely related to inpatient care utilization and unmet needs. Both inpatient visits and unmet needs were concentrated among respondents with multimorbidity and low EQ-VAS scores. Even if they had utilized more hospital services, they still had unmet needs. This finding confirmed the conclusion of a previous study that multimorbidity and poor self-rated health were risk factors for unmet needs [24, 38–40, 43]. Multimorbidity not only increases healthcare utilization and unmet needs but also increases the occurrence of catastrophic health expenditure for households and health inequity [44]. The widespread of multimorbidity in China has posed serious challenges to health systems and insurance schemes [45, 46]. To improve equitable access to healthcare, high-needs patients should be identified and targeted healthcare delivery systems should be developed in the future [45, 47].

Based on a representative survey, our study initially provides valuable evidence on equity in hospitalization visits and unmet needs among the Tibetan population, and examines the drivers of equity and reasons for unmet needs. This study has several limitations. First, it was based on two waves of cross-sectional surveys. We could not make causal inferences based on this cross-sectional survey. Second, inpatient care utilization, unmet needs, and household income were measured based on self-report, which may lead to an underestimation of bias in prevalence and equity. Third, the analysis of the reasons for unmet inpatient care needs was based on a structured questionnaire, which could only provide basic information. Detailed information on other reasons could not be obtained from the questionnaire. In the future, qualitative studies should be conducted to investigate the underlying causes and mechanisms, and to inform the design of acceptable and available telehealth care.

## Conclusions

Our findings suggest that inequity in inpatient care utilization narrowed from 2013 to 2018 and that there was no inequity in unmet needs in Tibet. The main reason for unmet needs shifted from financial hardship to other reasons, and financial affordability improved in 2013 and 2018. The income and the NRCMS were the two main facilitators of equity in healthcare utilization. However, the widespread of chronic diseases, especially multimorbidity, has posed great challenges to equal access to inpatient care. To better achieve the political goal of the Chinese government and Sustainable Development Goal 3, policymakers should develop healthcare needs assessment mechanisms and targeted healthcare delivery systems to provide available and accessible healthcare for high-needs patients in Tibet.

## Electronic supplementary material

Below is the link to the electronic supplementary material.

Supplementary Table 1. Economic development, health resource allocation and health expenditure in Tibet between 2010 and 2020

## Data Availability

The data used in this study are not publicly available due to the confidential policy but are available from the corresponding author on reasonable request.

## Abbreviations

CI: concentration index
EQ-VAS: EuroQol-visual analog scale
HI: horizontal inequity index
NHSS: National Health Services Survey
NRCMS: New Rural Cooperative Medical Scheme
UEBMI: Urban Employee Basic Medical Insurance
URBMI: Urban Resident Basic Medical Insurance
OOP: out-of-pocket
THE: total health expenditure
