# Supplementary material for: Income-related equity in inpatient care utilization and unmet needs between 2013 and 2018 in Tibet, China
Source: Int J Equity Health. 2023 May 10;22:85. doi: 10.1186/s12939-023-01889-4 (PMC10173530; doi:10.1186/s12939-023-01889-4)
Supplement: Supplementary file 1 — Supplementary Table 1. Economic development, health resource allocation and health expenditure in Tibet between 2010 and 2020 [file 12939_2023_1889_MOESM1_ESM.docx]

Supplementary Table 1. Economic development, health resource allocation and health expenditure in Tibet between 2010 and 2020

| Year | Tibet | | | |  | The whole country | | | |
| --- | --- | --- | --- | --- | --- | --- | --- | --- | --- |
|  | GDP per capita^#^ | Bad density | Physician density | Share of OOP in THE |  | GDP per capita^#^ | Bad density | Physician density | Share of OOP in THE |
| 2010 | 40874 | 3.01 | 1.52 | - |  | 56087 | 3.58 | 1.8 | 35.29 |
| 2011 | 43081 | 3.17 | 1.38 | - |  | 59116 | 3.84 | 1.82 | 34.77 |
| 2012 | 44201 | 2.72 | 1.31 | 7.4 |  | 60653 | 4.24 | 1.94 | 34.34 |
| 2013 | 45350 | 3.53 | 1.63 | 7.5 |  | 62230 | 4.55 | 2.04 | 33.88 |
| 2014 | 46257 | 3.75 | 1.76 | 6.47 |  | 63474 | 4.85 | 2.12 | 31.99 |
| 2015 | 46905 | 4.33 | 1.9 | 5.71 |  | 64363 | 5.11 | 2.22 | 29.27 |
| 2016 | 47843 | 4.37 | 1.98 | 5.46 |  | 65650 | 5.37 | 2.31 | 28.78 |
| 2017 | 48608 | 4.78 | 2.26 | 5.16 |  | 66700 | 5.72 | 2.44 | 28.77 |
| 2018 | 49629 | 4.88 | 2.42 | 5.62 |  | 68101 | 6.03 | 2.59 | 28.61 |
| 2019 | 51068 | 4.87 | 2.66 | 6.28 |  | 70076 | 6.30 | 2.77 | 28.36 |
| 2020 | 52345 | 4.32 | 2.59 | 7.16 |  | 71828 | 6.46 | 2.90 | 27.65 |

GDP, gross domestic product; OOP, out-of-pocket; THE, total health expenditure. -, unavailable.

^#^Numbers adjusted for inflation and measured in Chinese yuan in 2020 prices.
